# Protocol for a systematic review and meta-analysis on the effects of cold-water exposure on mental health

**DOI:** 10.3389/fpsyt.2025.1603700

**Published:** 2025-06-02

**Authors:** Steven Schepanski, Florian Batta, Marleen Schröter, Georg Seifert, Anna Katharina Koch

**Affiliations:** ^1^ Charité – Universitätsmedizin Berlin, Corporate Member of Freie Universität Berlin and Humboldt-Universität zu Berlin, Charité Competence Center for Traditional and Integrative Medicine (CCCTIM), Berlin, Germany; ^2^ Charité – Universitätsmedizin Berlin, Corporate Member of Freie Universität Berlin and Humboldt-Universität zu Berlin, Department of Pediatrics, Division of Oncology and Hematology, Berlin, Germany

**Keywords:** cold-water exposure, mental health, stress reduction, anxiety, depression, swimming-based, ice-bathing, cold-water immersion

## Abstract

Cold-water exposure has gained increasing popularity as a self-applied intervention for improving mental health and psychological well-being. While anecdotal evidence and popular media suggest potential benefits for reducing stress, anxiety, and depressive symptoms, the scientific evidence supporting these claims remains fragmented. This protocol outlines the methodology for a systematic review and meta-analysis aiming to critically appraise and synthesize the available evidence on the effects of CWE on mental health outcomes in adult populations, including both healthy individuals and those with clinical conditions. The review will examine both psychological variables and physiological stress markers in response to cold-water exposure interventions. Following PRISMA 2020 guidelines, a systematic search will be conducted in PubMed, PsycINFO, Embase, and Web of Science. Eligible studies will include randomized controlled trials, controlled clinical trials without randomization, cohort studies, and case-control studies. Risk of bias will be assessed using RoB1 for randomized controlled trials, ROBINS-I for non-randomized intervention studies, and ROBINS-E for observational studies. Random-effects meta-analyses will be conducted where sufficient data are available. The review will provide a structured quantitative synthesis of cold-water exposure’s effects on mental health, helping to bridge the gap between popular claims and empirical evidence. The findings will inform future research, public health guidelines, and the development of safe and effective cold-water exposure protocols for clinical and wellness applications.

## 1 Introduction

### 1.1 Background and conceptual framing

Cold-water exposure (CWE), including practices such as ice bathing, winter swimming, and cold showers, has gained increasing popularity in recent years as a wellness trend. Promoted for its physical and mental health benefits, CWE is rooted in a long tradition of hydrotherapy practices. Historical accounts describe its use by Hippocrates (e.g., 1, 2), and its popularization in the 19th century by Vincenz Prießnitz and Sebastian Kneipp led to its integration into naturopathic medicine (3). In modern contexts, CWE is used in athletic recovery (e.g., 4–7), leisure culture (8), and proposed as a mental health-promoting intervention (9, 10).

Mental health disorders, particularly anxiety and depressive disorders, are among the leading causes of disability worldwide and are associated with significant impairments in functioning, quality of life, and increased comorbidity risk (11). First-line treatments include pharmacotherapy and evidence-based psychotherapy; however, both approaches face important limitations. Antidepressants and anxiolytics, while effective, are frequently associated with side effects (12) such as fatigue (13), sexual dysfunction (14), or weight gain (15), and may be contraindicated in certain populations (16). Access to psychotherapy is often limited by availability, cost, or long waiting times, particularly in publicly funded health systems (17). Recent evidence suggests that certain non- pharmacological interventions, such as mindfulness-based stress reduction (MBSR), can achieve clinical outcomes comparable to pharmacological treatment. For instance, a randomized controlled trial demonstrated that MBSR was non-inferior to escitalopram in the treatment of anxiety disorders, with a potentially more favorable side effect profile (18). These findings underscore the growing interest in low-threshold, biologically plausible, and self-administered interventions. In this context, CWE may represent a promising complementary approach that warrants rigorous evaluation.

Physiologically, CWE activates multiple systems, including the autonomic nervous system and the hypothalamic-pituitary-adrenal (HPA) axis (19–22). Acute effects include sympathetic activation, endocrine changes (e.g., increased norepinephrine and cortisol), and respiratory responses such as hyperventilation (23). With repeated exposure, adaptive processes such as cross-stressor habituation (1, 24, 25) have been associated with upregulation of antioxidant defenses and reductions in oxidative stress markers (26, 27), as well as with modulation of inflammatory cytokines such as elevated basal IL-6 levels, potentially reflecting an adaptive immune activation, and attenuated IL-1β and IL6 release upon stimulation in habitual winter swimmers (21, 28). These may extend beyond physical regulation to influence psychological functioning, because some evidence suggests that CWE may reduce depressive symptoms (9), improve mood (29, 30), lower perceived stress (31), and enhance sleep and well-being (20, 32), therefore proposed mechanisms include neurotransmitter regulation and anti-inflammatory effects.

Another key mechanism underlying these effects may be CWE’s impact on the HPA axis and cross-adaptive stress responses. CWE acts as an acute physiological stressor, triggering activation of both the sympathetic nervous system and the HPA axis, leading to increased norepinephrine and epinephrine, as well as cortisol secretion as part of the acute stress response (23). Evidence suggests that repeated CWE may lead to a decreased cortisol response over time, indicating a potential adaptive effect that could improve overall stress resilience (21). The cross-adaptation hypothesis posits that repeated exposure to cold stressors may generalize to improved stress responses in other domains, such as emotional regulation and anxiety management (1, 24). By fostering a more balanced physiological stress response, CWE could play a role in mitigating the impact of chronic stress, which is a key factor in many mental health conditions.

Beyond its physiological effects, CWE may also hold promise as an intervention due to its influence on behavior and cognition. Interestingly, published evidence (33, 34) suggests that acute sympathetic arousal, such as that induced by CWE, may transiently enhance aspects of executive functioning, particularly response inhibition, while impairing others like working memory, depending on context and individual factors. Psychological mechanisms such as behavioral activation and enhanced self-efficacy may contribute to its mental health benefits. Overcoming the initial discomfort of cold exposure, specifically when guided by a gradual acclimation process, can serve as a reinforcing experience, potentially restoring sensitivity to positive reinforcement mechanisms that are often impaired in depression (10, 35). The sense of mastery and control gained from enduring cold stressors may enhance self-efficacy, which is closely linked to mental well-being and resilience (36). Additionally, the communal aspects of group-based CWE may foster positive social interactions and tackle feelings of deficiency and loneliness that are common among patients with mental disorders. Evidence from research on the efficacy of psychotherapy in group settings has identified group cohesion as an influential therapeutic factor next to interventions themselves (37). Despite its potential, these psychological pathways have received little attention in the literature, highlighting the need for further research to explore CWE as an adjunct therapeutic approach for populations with mental health disorders.

These observations align with existing psychoneurobiological models of mental health, which highlight dysfunction in stress response systems (e.g., HPA axis), altered immune signaling (e.g., IL-6 or TNF-α), and impaired cognitive performance as key mechanisms in conditions such as depression and anxiety (34, 38, 39). CWE may act on similar targets, suggesting a plausible mechanistic overlap with established treatments. For example, reduced cortisol reactivity and anti-inflammatory effects observed in habitual cold water users (21, 28) mirror physiological changes seen in response to antidepressant or psychotherapy (40, 41). By engaging both physiological and psychological mechanisms, CWE may offer a complementary pathway to restoring stress resilience and affect regulation in individuals with mood and anxiety disorders (42).

### 1.2 Previous reviews

Despite growing public enthusiasm and promising mechanisms, systematic attempts to synthesize the evidence on CWE remain limited and face notable methodological challenges. Most available studies focus primarily on physiological outcomes, are conducted in healthy populations, or vary widely in study design quality and intervention protocols (1, 6, 8, 43). A recent systematic review by Cain, Brinsley (44) evaluated the cognitive and physiological effects of CWE in healthy adults and included some psychological outcomes, however their meta-analysis was limited to randomized-controlled trials (RCTs), reducing the breadth of included evidence. Of 11 studies analyzed (N = 3,177 participants), only one-time reductions in stress were found 12 hours post-CWE, while no immediate, 1-hour, or long-term effects were observed. The review also found limited data on mood and depression due to a lack of well-controlled RCTs.

One key issue with the existing literature is the confounding influence of sports performance context, because many studies apply CWE in atheletic recovery settings, where the primary aim is to reduce inflammation or improve physiological recovery following acute exercise. While informative for sports science, these studies offer limited insights into CWE’s effects on mental health, as the inflammatory pathways activated by physical exercise, such as toll-like receptor (TLR) signaling and reactive oxygen species (ROS) generation, differ in their peripheral triggers and signaling cascades from those initiated by psychological stress (45). Cain, Brinsley (44), for example, included four studies that involved high-intensity or resistance training prior to CWE (46–49). Theoretically, even swimming-based CWE, although not structured as anaerobic training, can elicit anaerobic metabolism, due to reduced muscle oxygenation and increased cardiovascular stain in cold water. Earlier lactate accumulation and fatigue have been reported even at submaximal intensities in water temperature ≤25°C (e.g., 20, 28, 50). Thus, observed psychological effects in such settings cannot be attributed solely to cold exposure. For the purpose of our systematic review and meta-analysis, CWE is defined as immersion in water temperature ≤25°C, a range discussed in thermophysiological literature to be interpreted as “icy” (<12°C) and “cold” (12–24°C) (51, 52). However, we want to acknowledge that no universally accepted classification system for cold-water exposure currently exists, and temperature thresholds should be interpreted pragmatically.

Another persistent challenge is the heterogeneity in CWE protocols across studies, in which water temperature from 7–24°C, with immersion durations varying between 30 seconds and 2 hours and differing depths (e.g., full-body versus partial immersion). Previous reviews have either not addressed this variation analytically (e.g., 1, 43, 53, 54) or neglected to account for it in meta-analyses (e.g., 44). Moreover, the populations studies are often healthy and athletic, limiting generalizability to individuals with mental health conditions or more diverse background. There is also a lack of longitudinal studies assessing sustained psychological effects, even if some studies examined acute affective outcomes (e.g., 30, 55) or follow effects over several weeks or months (e.g., 20, 56), extended follow-ups in RCTs to determine sustained effects remain rare.

Finally, methodological limitations in risk of bias further challenge interpretation. Cain, Brinsley (44) used the PEDro scale, originally developed for physiotherapy and related health sciences (57), and reported a mean quality score of 6.4, indicating moderate quality, meaning some studies with high risk of bias were still included. In contrast to pharmacological trials, blinding in CWE studies is inherently difficult, as participants easily recognize whether they are exposed to cold water or not, which introduces expectancy effects and observer biases. Additionally, psychological outcomes such as well-being, stress, or symptoms are often self-reported, and only rarely triangulated with objective markers such as cortisol. These are inherent limitations in this field of research, and while rigorous critical appraisal is necessary, they cannot always be fully eliminated.

### 1.3 Rationale

A small but growing number of studies suggest that CWE may exert beneficial effects on mental health outcomes, including reductions in depressive symptoms, anxiety, and perceived stress. For example, a recent randomized trial compared a 3-week cold-water and breathing intervention based on the Wim Hof Method to an active control with warm showers and slow breathing in midlife women with high stress and depressive symptoms (58). Both groups showed significant and sustained improvements in depressive and anxiety symptoms, but the CWE group reported greater reductions in daily stress rumination. Similarly, another study conducted as a feasibility trial (59) of twice-weekly cold-water swimming as an add-on treatment for patients with clinically diagnosed depression. The intervention was well-tolerated and associated with improvements in subjective well-being and sleep quality.

While these findings are promising, the evidence base remains fragmented. The existing studies differ considerably in terms of population characteristics, study design, intervention parameters (e.g., water temperature, exposure frequency), and outcome measures. Sample sizes are often small, control conditions heterogeneous, and follow-up assessments limited. These factors restrict the generalizability and clinical interpretation of findings. A systematic review and meta-analysis are therefore warranted to synthesize available data across diverse study types, clarify under which conditions CWE may be effective, and guide future clinical applications and research directions.

## 2 Methods and analysis

To ensure methodological rigor and transparency, the protocol has been pre-registered with PROSPERO (Registration Number: CRD420250654531).

### 2.1 Objectives

#### 2.1.1 Primary objectives

The overarching aim of this systematic review and meta-analysis is to synthesize the effect of CWE on mental health outcomes in adult populations. Therefore, our primary objective is to assess the effects of CWE on key mental health outcomes, specifically depressive symptoms, anxiety, perceived stress, and general mental well-being, by pooling effect sizes from available studies using validated psychometric instruments.

#### 2.1.2 Secondary objectives

The secondary objectives are:

1. To investigate the effects of CWE on broader psychological and physiological variables associated with mental health, including sleep quality, thermoregulatory (e.g., core body temperature, skin temperature), metabolic responses, and neurobiological markers such as cortisol, norepinephrine, and inflammatory cytokines (e.g., IL-6, TNF-α or C-reactive protein (CRP)).
2. To compare the effects of CWE between healthy individuals and clinical populations from whom mental health outcomes are assessed.
3. To conduct subgroup analyses to examine whether the effects of CWE on psychological and physiological outcomes vary depending on intervention characteristics, including:
  - Type of exposure: full-body exposure versus chest-level exposure.
  - Frequency and duration of exposure: single versus repeated sessions.
  - Water temperature: <5°C, 5–11°C, 12–18°C, and 19-24°C (considering comparable immersion durations where possible).
  - Modality: active exposure such as swimming versus passive exposure such as bathing or showering.
  - Biological sex.
  - Body composition or BMI (e.g., normal versus elevated), where reported.
4. Dose-response relationships (e.g. minimum effective exposure duration or frequency required to achieve psychological benefits).
5. Long-term effects and sustainability of CWE benefits, defined as outcomes assessed four weeks or more after the final intervention session, and to identify adherence barriers and motivators for continued practice over time.
6. To summarize reported adverse events, safety concerns, and contraindications related to CWE, and to identify practical barriers to implementation in real-world settings.

### 2.2 Hypotheses

#### 2.2.1 Primary hypothesis

CWE has a statistically significant effect on key mental health outcomes, specifically, it reduces depressive symptoms, anxiety, and perceived stress, and improves general mental well-being, compared to no intervention or passive control condition.

#### 2.2.2 Secondary hypothesis

1. CWE improves sleep quality, modulates thermoregulatory responses (e.g., reduced core body temperature and skin temperature after exposure), influences metabolic processes, and modulates neurobiological markers associated with mental health, specifically reduces cortisol concentrations, increases norepinephrine levels, and reduces pro-inflammatory cytokines (e.g., IL-6, TNF-α), compared to control conditions.
2. The effect of CWE on mental health outcomes are more pronounced in clinical populations, defined as individuals with somatic or psychiatric conditions from whom mental health outcomes were assessed, compared to healthy individuals.
3. The effects of CWE on psychological (e.g., depressive symptoms, anxiety, perceived stress, well-being) and physiological outcomes (e.g., cortisol, HRV) vary depending on
  - Type of exposure, with full-body exposure yielding greater effects than chest-level exposure.
  - Frequency and duration, with repeated sessions yielding greater effects than single sessions.
  - Water temperature (with immersion durations kept comparable across studies where possible), hypothesizing that lower temperatures may produce stronger effects compared to higher cold exposures.
  - Modality, with active exposure showing different effects than passive exposure.
  - Sex, with different effect patterns in males versus females.
  - Body composition or BMI (e.g., normal versus elevated), potentially due to differences in thermoregulation, metabolic activity, or insulation.
4. A dose-response relationship exists, such that longer and/or more frequent CWE sessions are associated with greater improvements in mental health outcomes.
5. CWE has sustainable effects on mental health outcomes that persist for at least four weeks after the intervention. However, adherence to CWE protocols may decrease over time, particularly in interventions involving very cold temperatures (<5°C), due to discomfort or safety concerns.
6. CWE may be associated with adverse physiological or psychological effects, particularly in vulnerable populations such as individuals with cardiovascular risk or posttraumatic stress disorder. Barriers such as discomfort, access, or safety concerns may limit real-world implementation.

### 2.3 Criteria for considering studies

#### 2.3.1 Study designs

We will include empirical studies that quantitatively examine the relationship between CWE and mental health outcomes (**Table 1**). Eligible study designs are RCTs, controlled clinical trials without randomization (CCTs), cohort studies and case control studies.

**Table 1:** Inclusion and exclusion criteria (PICOS framework).

PICOS Elements	Inclusion Criteria	Exclusion Criteria
Population	• Adults (≥18 years) • Healthy or clinical populations • All demographics	• Children/adolescents (<18 years) • Elite athletes • Sauna or physical activity prior to CWE (Recovery context)
Intervention (Exposure)	• Full-body or chest-level CWE (≤25°C) • Ice baths, cold showers, recreational open-water winter swimming	• Partial immersion (e.g., foot baths) • CWE + other therapies (if not comparable between groups)
Comparison	• Any control (e.g., placebo, usual care) • Pre-post within-group	
Outcomes	• Mental health (depression, anxiety, stress, well-being, life satisfaction) • Cortisol, HRV • Sleep, immune markers • Adherence, psychosocial effects (e.g. isolated versus group-event)	
Study Design	• RCTs • CTs • Cohort studies • Case-control studies • Pre-post studies	• Insufficient intervention details • Cross-sectional • Case reports/series • Reviews • Commentaries

CWE, cold-water exposure; HRV, heart rate variability; RCTs, randomized controlled trials.

The following types of studies will be excluded:

- Cross-sectional studies,
- Case reports and case series.
- Reviews (systematic or narrative), meta-analyses, conference abstracts, commentaries, and opinion articles, because they do not provide original primary data suitable for quantitative synthesis.

#### 2.3.2 Population

We will include studies investigating adult population (≥18 years), with no restrictions based on sex, gender, ethnicity, or geographic region, including both healthy individuals and those with pre-existing mental or physical health impairments. We also exclude studies involving elite athlete populations, defined as individuals competing at national or international levels, academic teams or school teams (e.g., professional swimmers, triathletes), where CWE is primarily used as a sports recovery strategy (**Table 1**).

#### 2.3.3 Intervention (exposure)

This review will include studies evaluating structured CWE interventions or exposures, operationalized as full-body immersion including head immersion, or chest-level immersion at or above the xiphoid process with the head remaining above the water, conducted in water at or below 25°C, in accordance with recent attempts to define thermal thresholds for CWE (51, 52) (**Table 1**). Accepted modalities include but are not limited to a) ice-water exposure, b) cold showers, c) recreational open-water winter swimming (e.g., non-professional, self-directed or supervised practice, in indoor or outdoor environments), and d) cold-water exposure in naturally cold environments such as lakes, rivers, or the ocean.

Studies exclusively employing partial-body immersion, such as hydrotherapy foot baths or Kneipp water treatments, will not be considered. Similarly, studies that include CWE as part of a complex intervention will be excluded. No specific minimum exposure duration or frequency will be imposed, but eligible studies must clearly describe CWE parameters (e.g., water temperature, immersion depth, exposure time, and frequency). Both single-session (acute) and repeated-session (multi-day or multi-week) interventions will be eligible for inclusion. Studies that adopt physical activity or passive heating such as sauna exposure prior to CWE in their studies will be excluded.

#### 2.3.4 Outcomes

The primary outcomes of interest will be changes in mental health indicators following CWE interventions, including self-reported measures assessed by validated instruments (**Table 1**). The following list provides examples for widely utilized instruments; however, we do not limit ourselves to them because they all quantify similar latent structures:

- Depressive symptoms: Beck Depression Inventory (BDI-II; 60), Patient Health Questionnaire-9 (PHQ-9; 61), Hamilton Depression Rating Scale (HAM-D; 62), Center for Epidemiologic Studies Depression Scale (CES-D; 63).
- Anxiety symptoms: Generalized Anxiety Disorder Scale (GAD-7; 64), Hamilton Anxiety Rating Scale (HAM-A; 65), State-Trait Anxiety Inventory (STAI; 66), Visual Analogue Scale (VAS).
- Perceived stress: Perceived Stress Scale (PSS; 67), Depression Anxiety Stress Scales (DASS-21 or DASS-42; 68), VAS.
- General well-being: WHO-5 Well-Being Index (69), Short Form-36 (SF-36; 70), Positive and Negative Affect Schedule (PANAS; 71).
- Life satisfaction: Satisfaction With Life Scale (SWLS; 72), World Health Organization Quality of Life (WHOQOL)-BREF (WHOQOL 73), Temporal Satisfaction with Life Scale (TSWLS; 72).
- Mood profiles: Profile of Mood States (POMS (74)).

Additionally, physiological stress indicators will be treated as primary outcomes, including cortisol concentrations from various sources, heart rate variability (HRV), electrodermal activity (EDA) as a sensitive proxy of sympathetic nervous system activity, and vascular or autonomic parameters such as pulse waveform analysis or photoplethysmographic (PPG). However, we acknowledge that PPG signal especially when using infrared light, may be less reliable during cold exposure due to vasoconstriction and perfusion changes (75, 76). These limitations will be carefully considered during data interpretation. HRV outcomes will include standard time-domain measures (e.g., root mean square of successive RR interval differences (RMSSD), standard deviation of NN intervals (SDNN)) and frequency-domain measures (e.g., high-frequency, low-frequency components (77)), extracted as reported in eligible studies. Where available, central nervous system markers such as electroencephalogram (EEG) or functional magnetic resonance imaging (fMRI) will also be considered, as they provide insights into brain activity under stress and may reflect neurocognitive effects of CWE.

The secondary outcomes will include sleep-related parameters such as Pittsburgh Sleep Quality Index (PSQI; 78), neuroendocrine such as norepinephrine, and immune biomarkers such as IL-6 or TNF-α. In addition, we will extract and synthesize any reported adverse events, physiological or psychological complications, and safety-related recommendations across studies. This includes dropout reasons due to intolerance, acute somatic reactions (e.g., cold shock, dizziness), and psychological distress. These outcomes will help identify potential contraindications and inform practical considerations for safe implementation.

Exploratory outcomes will capture behavioral and psychosocial aspects such as adherence rates to CWE, participant-reported motivators and barriers, and contextual factors such as individual versus group-based CWE engagement.

### 2.4 Search strategy and data management

#### 2.4.1 Search strategy

We will implement a multi-stage, systematic search strategy to identify eligible studies. The search will be carried out across four electronic databases without date restrictions: MEDLINE via Ovid, PsycINFO, Embase, and Web of Science. Additionally, trial registries such as ClinicalTrials.gov and the WHO International Clinical Trials Registry Platform (ICTRP) will be searched to capture ongoing trials.

To increase the likelihood of capturing all relevant studies, we will also conduct backwards and forward citation tracking using the CitationChaser tool (79). This tool will help identify additional records from the reference lists of eligible articles and relevant systematic reviews not detected through database queries. Furthermore, a filter will be applied to limit the search to human studies only based on search string recommendations. Studies in all languages will be screened either by a native speaker or translated via AI (ChatGPT or DeepL) into English. For studies deemed potentially eligible, full translation and interpretation will involve a fluent speaker of the respective language, either from the review team or collaborating researchers. As a result, a comprehensive search string was developed in collaboration with an experienced information specialist and is as an example for Ovid MEDLINE available in **Supplementary Table 1**.

#### 2.4.2 Study selection

All retrieved records will be imported into Rayyan.ai (80) to facilitate the screening and deduplication process. The platform utilizes an automated similarity algorithm that compares elements such as article titles, author lists, and journal names. Records flagged with a similarity score of at least 95% will be classified as duplicates and excluded and for records with lower similarity but potential overlap, FB will conduct a manual inspection to confirm duplication status.

When studies are reported across multiple publications, such as interim reports or secondary analyses, we will consolidate data and extract information from the most complete or primary source to avoid double counting results. Any supplementary information provided in secondary publications will be noted as integrated where relevant. In cases where critical data are incomplete or unclear, corresponding authors will be contacted in total twice times by email, where each period between contact attempts lasts two weeks. All communication efforts will be documented.

The study selection will be conducted in two phases by independent reviewers.

- Phase 1 (Title and Abstract Screening): Two reviewers will independently screen all titles and abstracts to exclude irrelevant studies. Reviewer FB will always be the first reviewer, while the second reviewer will rotate between SS, MS, or AKK.
- Phase 2 (Full-Text Screening): Full texts of potentially relevant studies will be retrieved and reviewed in detail against the inclusion and exclusion criteria based on the same reviewer scheme.

Any disagreements during either phase will be resolved by consensus between the two reviewers and if no consensus can be reached, a third reviewer will adjudicate (rotating between SS, MS, or AKK depending on initial reviewer pairing). The entire selection process will be reported according to PRISMA 2020 guidelines (81), including a PRISMA flow diagram detailing records identified, screened, excluded (with reasons), and included in the final synthesis.

#### 2.4.3 Data extraction

Data will be extracted in duplicate using a customized extraction form developed for this review, which will be piloted on a random sample of two included studies to ensure usability and clarity, prior to formal data extraction.

The following core information will be collected:

- Study characteristics: record identifier, authors, publication year, country where the study was conducted, study design, language, funding information, and sample procedures.
- Participant details: sample size, mean/median age, sex distribution, health status (e.g., presence or absence of diagnosed mental health conditions), baseline mental health status (if available), and anthropometric indices (e.g., BMI, body fat percentage).
- Intervention characteristics: CWE modality (e.g., ice bath, cold shower), water temperature, session duration (minutes), frequency (e.g., daily, weekly), total number of sessions, immersion depth (e.g., chest-level versus full-body), setting (indoor versus outdoor), supervision, and group versus individual application. The intervention details will be extracted in accordance with the Template for Intervention Description and Replication (TIDieR; 82) checklist to ensure comprehensive reporting of the intervention components.
- Comparator group(s): type of comparator (e.g., no intervention, active control, pre-post comparison).
- Outcomes:
  - Psychological outcomes: depressive symptoms, anxiety, perceived stress, general well-being, life satisfaction assessed via validated instruments.
  - Physiological outcomes: cortisol concentration from various sources, neuroendocrine markers (e.g., norepinephrine, dopamine), inflammatory biomarkers (e.g., IL-6, TNF-α), HRV outcomes (e.g., RMSSD, SDNN, LF, HF components) as reported, EDA, vascular and autonomic parameters (e.g., pulse waveform analysis, PPG), thermoregulatory indicators such as core body temperature and skin temperature, central nervous system outcomes such as EEG and fMRI.
  - Adverse events and safety-related information: reported physiological (e.g., cold shock, dizziness, cardiovascular events) or psychological (e.g., panic reactions, dropouts due to distress) adverse events, contraindications noted by study authors, reasons for dropout, safety measures applied (e.g., medical screening, supervision), and any safety recommendations included in the studies.
- Statistical data: measures of central tendencies (mean, median, mode), measures of dispersion (standard deviations, variance, range, interquartile range), effect sizes, standard errors, 95% confidence intervals (CIs), and other relevant statistics for meta-analytic synthesis.

Discrepancies between reviewers will be discussed and resolved via consensus between the two reviewers, or referred to a third reviewer when needed.

### 2.5 Study quality and critical appraisal

#### 2.5.1 Quality assessment

For RCTs, the Cochrane Risk of Bias tool 1 (RoB 1; 83) is a standard instrument for assessing risk of bias and evaluates domains such as randomization, allocation concealment, blinding, missing data, and selective reporting, providing a robust framework (**Table 2**). We will apply the Risk of Bias in Non-Randomized Studies of Interventions (ROBINS-I; 84) tool to controlled clinical trials without randomization and to quasi-experimental designs, as this tool is specifically designed to assess non-randomized interventions, including biases arising from confounding, participant selection, intervention classification, and deviations from intended interventions. While ROBINS-I was originally developed for comparative designs, we treat pre-post studies as quasi-experimental interventions, where similar sources of bias can arise. Using ROBINS-I allows for a structured and consistent assessment of internal validity across all intervention studies in the review.

**Table 2:** Overview of critical appraisal tools.

Study Design	Tool	Reference
Randomized controlled trial	RoB 1	(83)
Controlled clinical trial without randomization (intervention assigned)	ROBINS-I	(84)
Pre-post intervention studies (single- or multi-arm)	ROBINS-I	(84)
Observational cohort and case-control studies (exposure-based, not assigned)	ROBINS-E	(85)

RoB 1, Cochrane Risk of Bias Tool 1; ROBINS-I, Risk of Bias in Non-Randomized Studies of Interventions (ROBINS-I); ROBINS-E, Risk of Bias in Non-Randomized Studies of Exposures.

For observational designs (e.g., cohort or case-control studies) where cold-water exposure occurs naturally and is not assigned by researchers, we will apply the ROBINS-E (85) tool, which is specifically designed to assess risk of bias in non-randomized studies of exposures (**Table 2**). It evaluates domains such as confounding, selection bias, and misclassification of exposure, missing data, and bias in outcome measurement and reporting.

The quality assessment will be conducted by two independent reviewers. The primary reviewer (FB) will assess all included studies, while the second reviewer will rotate between SS, MS, or AKK according to a predefined allocation schedule. Any discrepancies in risk of bias appraisal will be resolved through consensus discussions; if disagreements persist, a third reviewer (either SS, MS, or AKK) will be consulted for arbitration. To evaluate the consistency and reliability of the risk of bias assessments, interrater agreement will be quantified using Cohen’s κ statistic (86), with values interpreted as κ = 0.61–0.80 indicating substantial agreement and κ > 0.80 indicating almost perfect agreement (87). The results of the quality assessment will directly inform both the interpretation of findings and subsequent sensitivity analyses. Studies identified as having a high risk of bias may be flagged for further scrutiny and where appropriate, sensitivity analyses will be conducted to assess the influence of these studies on the overall pooled effect estimates.

Given the importance of safety-related outcomes in this review, we will also consider incomplete or absent reporting of adverse events or contraindications as a potential source of bias, particularly under the domains of selective outcome reporting.

### 2.6 Analysis

#### 2.6.1 Descriptive analysis

We will initially conduct a quantitative descriptive analysis to summarize the characteristics of all studies in the review. Descriptive statistics, such as means, medians, standard deviations, and interquartile ranges, will be used to describe key variables including study design, sample sizes, participant characteristics such as age, sex distribution, clinical status, and CWE intervention details such as modality, water temperature, immersion depth, session duration, and frequency of exposure, comparator types, and outcome variables.

We will also provide a summary of both psychological and physiological outcomes assessed, including the instruments used for that and identify patterns in how these outcomes are operationalized across studies. This synthesis will highlight common trends in intervention protocols and populations, while also identifying outliers or heterogeneity in methodologies (e.g., variability in CWE modalities or comparator groups). Both narrative summaries and graphical displays such as frequency, distributions, boxplots, and bar charts will be used to illustrate characteristics and trends across the included studies. All descriptive statistics and visualizations will be created using R (e.g., ‘tidyverse’ (88), ‘dplyr’ (89), ‘ggplot2’ (90)) or Python (e.g., ‘pandas’ (91), ‘matplotlib’ (92)), depending on project needs. The descriptive phase will also serve to identify clusters of studies with similar characteristics, which may guide subsequent subgroup and sensitivity analyses. FB will conduct this analysis in close collaboration with SS. All authors will interpret the results.

#### 2.6.2 Meta-analysis

Meta-analysis will be performed where ≥ 2 studies report comparable data for a given outcome domain. These will include:

- Psychological outcomes such as depression, anxiety, stress, well-being, and life satisfaction.
- Physiological outcomes such as cortisol, cytokines, and HRV.

In light of the emerging nature of this research area and the limited number of available studies (as reflected in previous reviews (44)), we consider a minimum of two studies a justified threshold for performing meta-analyses. While results from these small-scale syntheses will be interpreted with due caution, they may still offer valuable preliminary insights into potential effects and inform future research directions. Where findings fall near the lower bounds of interpretability, we will explicitly highlight the associated limitations.

Different outcome measures assessing the same latent construct (e.g., depressive symptoms, perceived stress) will be grouped within their respective domains based on theoretical and empirical overlap (e.g., BDI-II, PHQ-9, and CES-D will be synthesized under the construct of depression). For physiological outcomes, biomarkers representing the same stress axis (e.g., cortisol from blood or saliva) will be meta-analyzed together, where measurement units and timing are sufficiently comparable.

Continuous outcomes will be summarized using standardized mean differences (SMDs) calculated via Hedges’ g with 95% CIs. For dichotomous outcomes, we will use odds ratios (ORs) or risk ratios (RRs), depending on what is reported by the primary studies.

Given the expected heterogeneity across study designs, populations, and CWE protocols, all meta-analyses will employ random-effects models using Restricted Maximum Likelihood (REML) estimation (93). For analyses with very small number of studies (< 3), alternative estimators will be considered for sensitivity testing. Fixed-effects models will be used as additional sensitivity checks. To facilitate interpretation, SMDs will be categorized as small effects (SMD = 0.2), moderate effects (SMD = 0.5), and large effects (SMD ≥ 0.8). To explore whether variations in study design contribute to differences in effect sizes, we will conduct meta-regression analyses using study design as a categorical moderator which enables us to assess the extent to which methodological design impacts the pooled estimates. This approach accounts for variability both within and between studies, providing more generalizable estimates. Additionally, fixed-effects models will be run as part of sensitivity analyses to assess the robustness of the findings under different modeling assumptions.

Where appropriate, we may also explore Bayesian random-effects models to complement traditional REML estimation, particularly in cases of sparse data or substantial between-study heterogeneity. Bayesian models can offer more flexible uncertainty estimation and may provide additional insights where classical methods are limited. In meta-regression, we will explore study-level moderators such as year of publication and publication status, provided a sufficient number of studies is available to ensure model stability. These analyses will be treated as exploratory and interpreted cautiously.

For all meta-analyses, p-values < 0.05 will be considered statistically significant. However, given the multiple comparisons planned, we will apply the Benjamini-Hochberg False Discovery Rate (FDR) correction to control the expected proportion of Type I errors, ensuring that the conclusions drawn are robust despite multiple testing (94).

All meta-analytic computations will be conducted using R software, specifically the ‘meta’ (95), ‘metafor’ (93), and ‘robumeta’ (96) packages. These will allow for standard meta-analyses, advanced random-effects modeling, meta-regression, and robust variance estimation where appropriate. Should data synthesis not be possible due to insufficient data or substantial heterogeneity, we will present a structured narrative synthesis of the available evidence. This narrative synthesis will highlight patterns in the findings and contextualize results within the broader literature.

#### 2.6.3 Assessing heterogeneity

To assess heterogeneity across included studies, we will apply a range of complementary statistical measures. The I² statistic will be calculated to quantify the proportion of variability attributable to between-study heterogeneity rather than random error. In line with Cochrane Handbook guidance (97), values will be interpreted flexibly: 0–40% may indicate low heterogeneity, 30–60% may represent moderate heterogeneity, 50–90% may represent substantial heterogeneity, and values above 75% considerable heterogeneity. Interpretation will also consider the clinical relevance, direction, and magnitude of effect sizes in addition to statistical heterogeneity.

Additionally, Cochran’s Q statistic will be reported as a formal test of heterogeneity, with the understanding that Q is sensitive to the number of studies included. To further quantify between-study variance, tau-squared (τ²) will be estimated using the REML method, which offers a balance between bias and efficiency. While τ² does not have a standardized scale for interpretation, larger τ² values indicate greater unexplained variance across studies, and its magnitude will be interpreted in relation to the context of the included studies and the variability of their effect sizes.

To provide a clinically meaningful interpretation of heterogeneity, we will calculate 95% prediction intervals, offering insight into the range of true effects expected in similar future studies. In cases where substantial heterogeneity is detected (I² > 75%), potential sources will be explored through predefined subgroup and meta-regression analyses.

#### 2.6.4 Subgroup analyses

To address predefined hypotheses, the following subgroup analyses will be conducted, contingent on ≥ 2 studies per subgroup:

- CWE modality (cold showers, ice baths, winter swimming).
- Water temperature (<12°C, 12-18°C, and 19–24°C).
- Session duration (≤1 min versus >1 to ≤5 minutes versus >5 min).
- Exposure frequency (single vs. repeated).
- Population type (healthy versus clinical).
- Gender, for women the menstrual cycle if available.
- Study design (RCTs versus non-RCTs; studies with active controls vs. passive/no controls).
- Body composition or BMI.

We acknowledge that subgroup analyses based on small numbers of studies are limited in their statistical power and stability. In line with current methodological guidance (98), all subgroup results will be interpreted with caution. This approach balances methodological rigor with the goal of identifying potential effect modifiers in a still-developing field.

#### 2.6.5 Assessment of publication bias

Potential publication bias will be examined through several complementary approaches. First, funnel plots will be created to visually inspect for asymmetry, which may indicate small-study effects or other forms of bias. We will formally test for asymmetry using Egger’s regression test, with a significance threshold set a p < 0.05.

In cases where bias is suspected, we will apply Duval and Tweedie’s trim-and-fill method to adjust the pooled effect size for potential missing studies. Additionally, we will calculate Failsafe N, estimating how many unpublished studies with null findings would be required to render the overall results non-significant. Given criticisms regarding the overestimation of robustness in large meta-analyses using this method, we will interpret this statistic with caution.

If visual or statistical indications of bias remain, we will consider more advanced techniques such as p-curve analysis or the application of selection models to better assess the likelihood of selective reporting practices or publication bias. These methods will provide further insights into the distribution of significant findings and the robustness of the observed results.

## 3 Discussion

### 3.1 Impact of the review

This systematic review and meta-analysis aim to represent the most comprehensive synthesis to date on the psychological effects of CWE. By systematically evaluating existing studies, this work bridges the gap between widespread anecdotal claims about CWE’s mental health benefits and the current scientific evidence. The review contributes to the emerging field by identifying trends regarding the effects of CWE on stress, anxiety, depression, and overall well-being. Furthermore, the findings highlight patterns in intervention protocols (e.g., water temperature, immersion duration, frequency) that may inform future research and practical application in clinical and wellness contexts.

The results of this review may have practical utility in informing mental health interventions, particularly adjunctive strategies for stress reduction, anxiety relief, or mood improvement. Beyond clinical settings, the findings are relevant to broader wellness communities and to sport professionals seeking non-pharmacological methods to support psychological resilience. While elite athletes were excluded from this review, the synthesis nonetheless holds value for general populations engaged in wellness or recovery-oriented practices involving CWE.

From a policy perspective, this review may support the development of safe and evidence-based guidelines for CWE practice, considering both its potential psychological benefits and the physiological risks (e.g., cold shock, hypothermia, cardiovascular strain) associated with exposure. Additionally, the findings could influence future funding priorities by highlighting CWE as a promising intervention area within public mental health strategies and integrative therapies.

### 3.2 Challenges and limitations

A key limitation of this review is the considerable variability in CWE intervention protocols across studies, including differences in water temperature, immersion depth, session duration, environment (indoor, outdoor) and frequency. These inconsistencies make direct comparisons challenging. Furthermore, the mental health outcomes assessed across studies vary widely, encompassing both self-reported questionnaires and physiological stress markers such as cortisol, which complicates data synthesis due to measurement heterogeneity.

Moreover, reliable differentiation between symptom severity grades (e.g., mild, moderate, severe) across studies is not feasible due to substantial variation in the instruments used. While some psychometric tools (e.g., PHQ-9, HAM-D) are designed for clinical grading, others (e.g., CES-D, PANAS) are more research-focused and lack harmonized cut-offs. These tools differ in scale construction, sensitivity, and intended application, making it difficult to compare severity levels across studies. Additionally, most studies do not stratify results by baseline severity or report diagnostic thresholds, therefore, a structured meta-analytic investigation of severity effects is not planned, as it would likely yield unreliable results at this stage. We will, however, document available baseline scores and clinical classifications where provided and, if applicable, address trends descriptively in the narrative synthesis.

Although included psychometric instruments are grouped according to shared latent constructs such as depression or anxiety, variations in item content and validation contexts may introduce measurement heterogeneity. Differences in the emphasis on cognitive or affective symptom dimensions across instruments could influence the comparability of effect sizes. We will account for this variability when interpreting meta-analytic results, and sensitivity analyses may be considered if sufficient data allow.

The evidence base is largely dominated by studies involving healthy or non-clinical populations. This restricts the generalizability of findings to individuals with diagnosed psychiatric conditions such as major depressive disorder or generalized anxiety disorder, where physiological and psychological stress responses to CWE may differ. In addition, in these types of studies, blinding is absent, as participants are typically aware of their exposure to cold water, potentially influencing psychological outcomes via expectancy effects. Management these represents one major challenge when designing studies using self-reported outcome measures.

Additionally, studies involving pool or natural body immersion often require participants to be competent swimmers, which may result in the systematic exclusion of non-swimming individuals. This may further limit the generalizability of findings. However, our review includes non-swimming CWE modalities, such as cold showers and ice baths, which are accessible to broader populations and not dependent on swimming skills. These modalities are important for ensuring inclusivity in both research and real-world applications. During data extraction, we will document whether swimming ability was a requirement and consider its implications in the synthesis of results.

A further conceptual limitation lies in the absence of a universally accepted classification system for CWE. While thresholds such as ≤15°C have been commonly used in earlier reviews (44, 99, 100), these are not based on standardized thermoregulatory or perceptual criteria. Recent attempts to integrate physiological and subjective response (52) have proposed broader classification bands (e.g., <12°C as “icy”, 12–24°C as “cold”), but these may evolve with future empirical research further. In light of this, our review adopts a pragmatic threshold of (<25°C) to enhance ecological validity and inclusiveness, while also acknowledging the current lack of consensus in this field.

The potential for publication bias cannot be fully excluded, as studies reporting positive or significant findings may be more likely to be published. While funnel plot and Egger’s test were employed to assess and adjust for bias, these methods cannot entirely rule out the risk of selective reporting.

Although this review synthesizes only previously published data, the nature of the included outcomes, such as depressive symptoms, stress-related information, and other psychiatric constructs, raises important ethical considerations for responsible secondary analysis. In particular, we rely on the assumption that primary studies followed robust data protection protocols and obtained appropriate informed consent. However, such procedures are not always transparently reported, and their heterogeneity represents an inherent limitation in meta-analytic work. Based on recent guidance (101), we recognize the value of a tiered sensitivity model that distinguishes between highly sensitive (e.g., raw psychiatric symptom data), moderately sensitive (e.g., de-identified group-level summaries), and minimally sensitive data. While the published data used here fall into the latter two categories, any future effort to obtain additional data from study authors will be guided by this framework. We will request only fully anonymized datasets and ensure that any reuse aligns with the ethical approvals and consent procedures of the original study. This underscores a broader challenge in psychiatric meta-analyses, namely, the ethical dependence on upstream data collection practices that cannot be re-evaluated retrospectively. As open science practices expand, we advocate for transparency, robust consent models, and privacy-aware data sharing to protect participant dignity throughout the research lifecycle.

While some studies may report acute changes in physiological stress markers such as cortisol, norepinephrine, and inflammatory cytokines following CWE, the long-term effects of repeated exposure on these biomarkers remain insufficiently understood. This gap underscores the need for more longitudinal studies examining sustained physiological adaptations.

### 3.3 Methodological choices

To maintain conceptual clarity and methodological rigor, the review applied strict inclusion criteria to focus exclusively on studies investigating systematic CWE, excluding interventions such as whole-body cryotherapy or mixed-modality protocols. This approach enhances the specificity of the review.

The decision to include RCTs, quasi-experimental trials, and observational studies allowed for a more comprehensive mapping of the current evidence landscape, despite introducing additional heterogeneity. This inclusive approach reflects the still nascent and interdisciplinary nature of CWE research.

To ensure methodological rigor, risk of bias was assessed using tools tailored to the respective study designs. This multi-tool approach allowed for nuanced evaluation of study quality. The adoption of random-effects models in all meta-analyses was chosen to appropriately account for between-study variability, thus improving the generalizability of the results. Furthermore, meta-regression and subgroup analyses will be applied to explore potential moderators of CWE effects, including study-level characteristics such as publication year. In addition, participant characteristics such as age will be considered in the interpretation of findings, particularly in relation to potential variability in psychological and physiological responses to CWE. Sensitivity analyses, including exclusion of high-risk-bias studies and comparison of random- versus fixed-effects models, were conducted to assess the robustness of the findings.

## 4 Ethics and dissemination

### 4.1 Ethics

CWE interventions carry inherent physiological risks, particularly in vulnerable populations. Exposure to cold water can trigger acute responses such as cold shock, hypothermia, or cardiovascular events, especially in individuals with pre-existing cardiac conditions. Therefore, any clinical or research application of CWE should include a thorough medical screening process to identify individuals who may be at heightened risk. Ensuring safety through appropriate exclusion criteria and supervised protocols is essential to minimize the potential for harm.

CWE may elicit strong stress-related physiological responses, which could be contraindicated for certain psychological profiles. Specifically, individuals with post-traumatic stress disorder (PTSD), panic disorder, or heightened anxiety sensitivity may experience exacerbation of symptoms when exposed to the acute stress associated with cold exposure. Such adverse psychological reactions highlight the need for careful screening and informed consent, as well as for providing participants with the option to discontinue exposure at any point.

Several practical barriers may limit the widespread implementation of CWE-based interventions in mental health care settings. First, accessibility may be constrained, as not all individuals or communities have access to safe open-water environments or cold immersion facilities. Second, adherence could be a challenge, as maintaining a regular schedule of cold exposure may prove difficult for participants due to discomfort or logistical constraints, potentially reducing the long-term feasibility of such interventions. Lastly, the current lack of standardized protocols, regarding optimal water temperature, immersion depth, duration, and frequency, limits the ability to develop clear and evidence-based guidelines for clinical or wellness applications. Further research is needed to establish best-practice recommendations that ensure both efficacy and safety across diverse settings.

To address this, our article will also synthesize reported adverse events and contraindications, as well as implementation barriers such as access, adherence, and safety concerns. These findings will help contextualize the evidence and provide preliminary guidance for safe and feasible application of CWE in non-athletic populations.

In addition to physiological and psychological safety, ethical considerations also extend to data privacy in mental health research (101). While this review will analyze published data, we recognize the heightened sensitivity of psychiatric outcomes and the importance of responsible secondary use. Where additional data are requested from authors, we will ensure that only fully anonymized information is used and that its reuse aligns with the original study’s ethical approvals and consent procedures.

### 4.2 Dissemination

The findings of this systematic review and meta-analysis will be submitted to a peer-reviewed journal specializing in mental health, psychophysiology, and/or integrative health interventions. In addition, results will be presented at relevant national and/or international conferences focused on stress research, mental health interventions, and psychoneuroendocrinology to engage with academic and clinical audiences.

To enhance knowledge translation, the findings will also be disseminated to a broader audience through science communication platforms, including social media channels, blogs and/or public outreach initiatives. The goal is to bridge the gap between research and practice by making evidence accessible to the public and relevant stakeholders.

The results of this review will provide valuable insights for clinicians, mental health therapists, sports professionals, and wellness practitioners interested in incorporating CWE as part of stress reduction or mood management interventions. Moreover, the findings will inform the design of future intervention studies aiming to evaluate the feasibility and effectiveness of CWE for improving psychological well-being, reducing anxiety, and supporting stress management in both clinical and non-clinical populations.

## References

[1] Tipton MJ Collier N Massey H Corbett J Harper M . Cold water immersion: kill or cure? Exp Physiol. (2017) 102:1335–55. doi: 10.1113/EP086283 28833689

[2] Tsoucalas G Sgantzos M Karamanou M Gritzalis K Androutsos G . Hydrotherapy: Historical landmarks of a cure all remedy. Arch Balkan Med Union. (2015) 50:430–2.

[3] Steward J . The culture of the water cure in nineteenth-century Austria, 1800-1914. In: Anderson SC Tabb BH , editors. Water, leisure and culture European historical perspectives. Leisure, consumption, and culture. Bloomsbury Publishing, London (2002). p. 23–35.

[4] Crowther F Sealey R Crowe M Edwards A Halson S . Team sport athletes’ perceptions and use of recovery strategies: a mixed-methods survey study. BMC Sports Science Med Rehabilitation. (2017) 9:6. doi: 10.1186/s13102-017-0071-3 PMC5326499 28250934

[5] Costello JT Donnelly AE . Effects of cold water immersion on knee joint position sense in healthy volunteers. J sports Sci. (2011) 29:449–56. doi: 10.1080/02640414.2010.544047 21279863

[6] Petersen AC Fyfe JJ . Post-exercise cold water immersion effects on physiological adaptations to resistance training and the underlying mechanisms in skeletal muscle: A narrative review. Front Sports Act Living. (2021) 3:660291. doi: 10.3389/fspor.2021.660291 33898988 PMC8060572

[7] Periard JD Racinais S Timpka T Dahlstrom O Spreco A Jacobsson J . Strategies and factors associated with preparing for competing in the heat: a cohort study at the 2015 IAAF World Athletics Championships. Br J Sports Med. (2017) 51:264–70. doi: 10.1136/bjsports-2016-096579 PMC5318647 27815238

[8] Knechtle B Waśkiewicz Z Sousa CV Hill L Nikolaidis PT . Cold water swimming-benefits and risks: A narrative review. Int J Environ Res Public Health. (2020) 17(23):8984. doi: 10.3390/ijerph17238984 33276648 PMC7730683

[9] Hjorth P Rasmussen MRV . Cold water swimming as an add-on treatment for depression: a feasibility study. Eur Psychiatry. (2024) 67:S533–S4. doi: 10.1192/j.eurpsy.2024.1109 37381680

[10] van Tulleken C Tipton M Massey H Harper CM . Open water swimming as a treatment for major depressive disorder. Case Reports. (2018) 2018:bcr–2018-225007. doi: 10.1136/bcr-2018-225007 PMC6112379 30131418

[11] Friedrich MJ . Depression is the leading cause of disability around the world. JAMA. (2017) 317:1517–. doi: 10.1001/jama.2017.3826 28418490

[12] Trindade E Menon D Topfer L-A Coloma C . Adverse effects associated with selective serotonin reuptake inhibitors and tricyclic antidepressants: a meta-analysis. Cmaj. (1998) 159:1245–52. PMC1229819 9861221

[13] Papakostas GI Nutt DJ Hallett LA Tucker VL Krishen A Fava M . Resolution of sleepiness and fatigue in major depressive disorder: a comparison of bupropion and the selective serotonin reuptake inhibitors. Biol Psychiatry. (2006) 60:1350–5. doi: 10.1016/j.biopsych.2006.06.015 16934768

[14] Higgins A Nash M Lynch AM . Antidepressant-associated sexual dysfunction: impact, effects, and treatment. Drug Healthc Patient Saf. (2010) 2:141–50. doi: 10.2147/DHPS.S7634 PMC3108697 21701626

[15] Papakostas GI . Limitations of contemporary antidepressants: tolerability. J Clin Psychiatry. (2007) 68:11. doi: 10.1016/j.biopsych.2006.06.015 17900204

[16] Campos AI Mulcahy A Thorp JG Wray NR Byrne EM Lind PA . Understanding genetic risk factors for common side effects of antidepressant medications. Commun Med (Lond). (2021) 1:45. doi: 10.1038/s43856-021-00046-8 35602235 PMC9053224

[17] Coombs NC Meriwether WE Caringi J Newcomer SR . Barriers to healthcare access among U.S. adults with mental health challenges: A population-based study. SSM Popul Health. (2021) 15:100847. doi: 10.1016/j.ssmph.2021.100847 34179332 PMC8214217

[18] Hoge EA Bui E Mete M Dutton MA Baker AW Simon NM . Mindfulness-based stress reduction vs escitalopram for the treatment of adults with anxiety disorders: a randomized clinical trial. JAMA Psychiatry. (2023) 80:13–21. doi: 10.1001/jamapsychiatry.2022.3679 36350591 PMC9647561

[19] Datta A Tipton M . Respiratory responses to cold water immersion: neural pathways, interactions, and clinical consequences awake and asleep. J Appl Physiol. (2006) 100:2057–64. doi: 10.1152/japplphysiol.01201.2005 16714416

[20] Huttunen P Kokko L Ylijukuri V . Winter swimming improves general well-being. Int J circumpolar Health. (2004) 63:140–4. doi: 10.3402/ijch.v63i2.17700 15253480

[21] Leppäluoto J Westerlund T Huttunen P Oksa J Smolander J Dugué B . Effects of long-term whole-body cold exposures on plasma concentrations of ACTH, beta-endorphin, cortisol, catecholamines and cytokines in healthy females. Scandinavian J Clin Lab investigation. (2008) 68:145–53. doi: 10.1080/00365510701516350 18382932

[22] Huttunen P Rintamäki H Hirvonen J . Effect of regular winter swimming on the activity of the sympathoadrenal system before and after a single cold water immersion. Int J Circumpolar Health. (2001) 60:400–6. doi: 10.1080/22423982.2001.12113043 11590880

[23] Tipton MJ . The initial responses to cold-water immersion in man. Clin science. (1989) 77:581–8. doi: 10.1042/cs0770581 2691172

[24] Lunt HC Barwood MJ Corbett J Tipton MJ . ‘Cross-adaptation’: habituation to short repeated cold-water immersions affects the response to acute hypoxia in humans. J Physiol. (2010) 588:3605–13. doi: 10.1113/jphysiol.2010.193458 PMC2988521 20643773

[25] Adolph EF . General and specific characteristics of physiological adaptations. Am J Physiol. (1956) 184:18–28. doi: 10.1152/ajplegacy.1955.184.1.18 13283084

[26] Kralova Lesna I Rychlikova J Vavrova L Vybiral S . Could human cold adaptation decrease the risk of cardiovascular disease? J thermal Biol. (2015) 52:192–8. doi: 10.1016/j.jtherbio.2015.07.007 26267514

[27] Wesołowski R Mila-Kierzenkowska C Pawłowska M Szewczyk-Golec K Saletnik Ł Sutkowy P . The influence of winter swimming on oxidative stress indicators in the blood of healthy males. Metabolites. (2023) 13(2):143. doi: 10.3390/metabo13020143 36837762 PMC9967992

[28] Dugué B Leppänen E . Adaptation related to cytokines in man: effects of regular swimming in ice-cold water. Clin Physiol (Oxford England). (2000) 20:114–21. doi: 10.1046/j.1365-2281.2000.00235.x 10735978

[29] Kelly JS Bird E . Improved mood following a single immersion in cold water. Lifestyle Medicine. (2022) 3:e53. doi: 10.1002/lim2.v3.1

[30] Reed EL Chapman CL Whittman EK Park TE Larson EA Kaiser BW . Cardiovascular and mood responses to an acute bout of cold water immersion. J thermal Biol. (2023) 118:103727. doi: 10.1016/j.jtherbio.2023.103727 PMC10842018 37866096

[31] Kopplin CS Rosenthal L . The positive effects of combined breathing techniques and cold exposure on perceived stress: a randomised trial. Curr Psychol. (2022), 1–13. doi: 10.1007/s12144-022-03739-y PMC9540300 36248220

[32] Knill-Jones J Shadwell G Hurst HT Mawhinney C Sinclair JK Allan R . Influence of acute and chronic therapeutic cooling on cognitive performance and well-being. Physiol Behav. (2025) 289:114728. doi: 10.1016/j.physbeh.2024.114728 39515683

[33] Starcke K Wiesen C Trotzke P Brand M . Effects of acute laboratory stress on executive functions. Front Psychol. (2016) 7:461. doi: 10.3389/fpsyg.2016.00461 27065926 PMC4814494

[34] Shields GS Sazma MA Yonelinas AP . The effects of acute stress on core executive functions: A meta-analysis and comparison with cortisol. Neurosci Biobehavioral Rev. (2016) 68:651–68. doi: 10.1016/j.neubiorev.2016.06.038 PMC5003767 27371161

[35] Lewinsohn PM . A behavioral approach to depression. In: The psychology of depression: Contemporary theory and research. John Wiley & Sons, Oxford, England (1974). p. xvii,318–xvii.

[36] Bandura A . Self-efficacy mechanism in human agency. Am Psychol. (1982) 37:122. doi: 10.1037/0003-066X.37.2.122

[37] Burlingame GM McClendon DT Yang C . Cohesion in group therapy: A meta-analysis. Psychother (Chic). (2018) 55:384–98. doi: 10.1037/pst0000173 30335452

[38] Slavich GM Irwin MR . From stress to inflammation and major depressive disorder: a social signal transduction theory of depression. psychol bulletin. (2014) 140:774. doi: 10.1037/a0035302 PMC4006295 24417575

[39] Hodes GE Kana V Menard C Merad M Russo SJ . Neuroimmune mechanisms of depression. Nat Neurosci. (2015) 18:1386–93. doi: 10.1038/nn.4113 PMC4843114 26404713

[40] Pariante CM Lightman SL . The HPA axis in major depression: classical theories and new developments. Trends neurosciences. (2008) 31:464–8. doi: 10.1016/j.tins.2008.06.006 18675469

[41] Miller AH Maletic V Raison CL . Inflammation and its discontents: the role of cytokines in the pathophysiology of major depression. Biol Psychiatry. (2009) 65:732–41. doi: 10.1016/j.biopsych.2008.11.029 PMC2680424 19150053

[42] Southwick SM Charney DS . The science of resilience: implications for the prevention and treatment of depression. Science. (2012) 338:79–82. doi: 10.1126/science.1222942 23042887

[43] Esperland D Weerd L Mercer JB . Health effects of voluntary exposure to cold water - a continuing subject of debate. Int J circumpolar Health. (2022) 81:2111789. doi: 10.1080/22423982.2022.2111789 36137565 PMC9518606

[44] Cain T Brinsley J Bennett H Nelson M Maher C Singh B . Effects of cold-water immersion on health and wellbeing: A systematic review and meta-analysis. PloS One. (2025) 20:e0317615. doi: 10.1371/journal.pone.0317615 39879231 PMC11778651

[45] Allen J Sun Y Woods JA . Chapter fourteen - exercise and the regulation of inflammatory responses. In: Bouchard C , editor. Progress in Molecular Biology and Translational Science, vol. 135. Cambridge, Massachusetts: Academic Press (2015). p. 337–54. 10.1016/bs.pmbts.2015.07.003 26477921

[46] Earp JE Hatfield DL Sherman A Lee EC Kraemer WJ . Cold-water immersion blunts and delays increases in circulating testosterone and cytokines post-resistance exercise. Eur J Appl Physiol. (2019) 119:1901–7. doi: 10.1007/s00421-019-04178-7 31222379

[47] Ahokas EK Kyröläinen H Mero A Walker S Hanstock HG Ihalainen JK . Water immersion methods do not alter muscle damage and inflammation biomarkers after high-intensity sprinting and jumping exercise. Eur J Appl Physiol. (2020) 120:2625–34. doi: 10.1007/s00421-020-04481-8 PMC7674333 32880050

[48] Hironaga I Nobuhiko E Hideya NAKATSUKA NY . Effects of water immersion on mucosal immune defense after acute resistance exercise. Adv Exercise sports Physiol. (2019) 25:15–20.

[49] Roberts LA Nosaka K Coombes JS Peake JM . Cold water immersion enhances recovery of submaximal muscle function after resistance exercise. Am J Physiol Regulatory Integr Comp Physiol. (2014) 307:R998–R1008. doi: 10.1152/ajpregu.00180.2014 25121612

[50] Loria P Ottoboni S Michelazzi L Giuria R Ghisellini P Rando C . Salivary cortisol in an extreme non-competitive sport exercise: winter swimming. Natural Science. (2014) 06:387–98. doi: 10.4236/ns.2014.66039

[51] Ntoumani M Dugué B Rivas E Gongaki K . Thermoregulation and thermal sensation during whole-body water immersion at different water temperatures in healthy individuals: a scoping review. J thermal Biol. (2023) 112:103430. doi: 10.1016/j.jtherbio.2022.103430 36796887

[52] Ntoumani M Soultanakis H Rivas E Dugué B Potter AW Yermakova I . An integrated thermal sensation scale for estimating thermal strain in water. Med Hypotheses. (2024) 187:111342. doi: 10.1016/j.mehy.2024.111342

[53] Kunutsor SK Lehoczki A Laukkanen JA . The untapped potential of cold water therapy as part of a lifestyle intervention for promoting healthy aging. Geroscience. (2025) 47:387–407. doi: 10.1007/s11357-024-01295-w 39078461 PMC11872954

[54] Stachyrak K Gregułą A Mazur B Mika D Kłos A Turek K . A comprehensive review on the latest insights into cold therapies and their impact on the human body, with a focus on neurophysiological responses. J Education Health Sport. (2024) 58:154–75. doi: 10.12775/JEHS.2024.58.012

[55] Yankouskaya A Williamson R Stacey C Totman JJ Massey H . Short-term head-out whole-body cold-water immersion facilitates positive affect and increases interaction between large-scale brain networks. Biology. (2023) 12:211. doi: 10.3390/biology12020211 36829490 PMC9953392

[56] Lindeman S Hirvonen J Joukamaa M . Neurotic psychopathology and alexithymia among winter swimmers and controls–a prospective study. Int J Circumpolar Health. (2002) 61:123–30. doi: 10.3402/ijch.v61i2.174445 12078959

[57] Maher CG Sherrington C Herbert RD Moseley AM Elkins M . Reliability of the PEDro scale for rating quality of randomized controlled trials. Phys Ther. (2003) 83:713–21. doi: 10.1093/ptj/83.8.713 12882612

[58] Blades R Mendes WB Don BP Mayer SE Dileo R O’Bryan J . A randomized controlled clinical trial of a Wim Hof Method intervention in women with high depressive symptoms. Compr psychoneuroendocrinology. (2024) 20:100272. doi: 10.1016/j.cpnec.2024.100272 PMC11599992 39606690

[59] Hjorth P Sikjær MG Løkke A Jørgensen AM Jørgensen N Kaasgaard DM . Cold water swimming as an add-on treatment for depression: a feasibility study. Nordic J Psychiatry. (2023) 77:706–11. doi: 10.1080/08039488.2023.2228290 37381680

[60] Beck AT Steer RA Brown GK . BDI-II: Beck Depression Inventory Manual. 2nd ed. San Antonio: Psychological Corporation. (1996).

[61] Kroenke K Spitzer RL Williams JB . The PHQ-9: validity of a brief depression severity measure. J Gen Intern Med. (2001) 16:606–13. doi: 10.1046/j.1525-1497.2001.016009606.x PMC1495268 11556941

[62] Hamilton M . A rating scale for depression. J Neurol Neurosurg Psychiatry. (1960) 23:56–62. doi: 10.1136/jnnp.23.1.56 14399272 PMC495331

[63] Radloff LS . The CES-D Scale: A self-report depression scale for research in the general population. Appl psychol Measurement. (1977) 1:385–401. doi: 10.1177/014662167700100306

[64] Spitzer RL Kroenke K Williams JB Löwe B . A brief measure for assessing generalized anxiety disorder: the GAD-7. Arch Intern Med. (2006) 166:1092–7. doi: 10.1001/archinte.166.10.1092 16717171

[65] Hamilton M . The assessment of anxiety states by rating. Br J Med Psychol. (1959) 32:50–5. doi: 10.1111/j.2044-8341.1959.tb00467.x 13638508

[66] Spielberger CD . State-trait anxiety inventory for adults. (1983).

[67] Cohen S Kamarck T Mermelstein R . A global measure of perceived stress. J Health Soc Behavior. (1983) 24:385–96. doi: 10.2307/2136404 6668417

[68] Lovibond PF Lovibond SH . The structure of negative emotional states: comparison of the Depression Anxiety Stress Scales (DASS) with the Beck Depression and Anxiety Inventories. Behav Res Ther. (1995) 33:335–43. doi: 10.1016/0005-7967(94)00075-U 7726811

[69] Topp CW Østergaard SD Søndergaard S Bech P . The WHO-5 Well-Being Index: a systematic review of the literature. Psychother Psychosom. (2015) 84:167–76. doi: 10.1159/000376585 25831962

[70] Jenkinson C Coulter A Wright L . Short form 36 (SF36) health survey questionnaire: normative data for adults of working age. Bmj. (1993) 306:1437–40. doi: 10.1136/bmj.306.6890.1437 PMC1677870 8518639

[71] Watson D Clark LA Tellegen A . Development and validation of brief measures of positive and negative affect: The PANAS scales. J Pers Soc Psychol. (1988) 54:1063–70. doi: 10.1037/0022-3514.54.6.1063 3397865

[72] Pavot W Diener E Suh E . The temporal satisfaction with life scale. J Pers Assessment. (1998) 70:340–54. doi: 10.1207/s15327752jpa7002_11

[73] Group W . Development of the World Health Organization WHOQOL-BREF quality of life assessment. The WHOQOL Group. Psychol Med. (1998) 28:551–8. doi: 10.1017/S0033291798006667 9626712

[74] Curran SL Andrykowski MA Studts JL . Short form of the profile of mood states (POMS-SF): psychometric information. psychol assessment. (1995) 7:80. doi: 10.1037/1040-3590.7.1.80

[75] Maeda Y Sekine M Tamura T . The advantages of wearable green reflected photoplethysmography. J Med Syst. (2011) 35:829–34. doi: 10.1007/s10916-010-9506-z 20703690

[76] Allen J . Photoplethysmography and its application in clinical physiological measurement. Physiol Meas. (2007) 28:R1–39. doi: 10.1088/0967-3334/28/3/R01 17322588

[77] Shaffer F Ginsberg JP . An overview of heart rate variability metrics and norms. Front Public Health. (2017) 5:258. doi: 10.3389/fpubh.2017.00258 29034226 PMC5624990

[78] Buysse DJ Reynolds CF 3rd Monk TH Berman SR Kupfer DJ . The Pittsburgh Sleep Quality Index: a new instrument for psychiatric practice and research. Psychiatry Res. (1989) 28:193–213. doi: 10.1016/0165-1781(89)90047-4 2748771

[79] Haddaway NR Grainger MJ Gray CT . Citationchaser: A tool for transparent and efficient forward and backward citation chasing in systematic searching. Res Synth Methods. (2022) 13:533–45. doi: 10.1002/jrsm.v13.4 35472127

[80] Ouzzani M Hammady H Fedorowicz Z Elmagarmid A . Rayyan—a web and mobile app for systematic reviews. Systematic Rev. (2016) 5:210. doi: 10.1186/s13643-016-0384-4 PMC5139140 27919275

[81] Page MJ McKenzie JE Bossuyt PM Boutron I Hoffmann TC Mulrow CD . The PRISMA 2020 statement: an updated guideline for reporting systematic reviews. BMJ. (2021) 372:n71. doi: 10.1136/bmj.n71 33782057 PMC8005924

[82] Hoffmann TC Glasziou PP Boutron I Milne R Perera R Moher D . Better reporting of interventions: template for intervention description and replication (TIDieR) checklist and guide. Bmj. (2014) 348:g1687. doi: 10.1136/bmj.g1687 24609605

[83] Higgins JPT Altman DG Gøtzsche PC Jüni P Moher D Oxman AD . The Cochrane Collaboration’s tool for assessing risk of bias in randomised trials. BMJ. (2011) 343:d5928. doi: 10.1136/bmj.d5928 22008217 PMC3196245

[84] Sterne JA Hernán MA Reeves BC Savović J Berkman ND Viswanathan M . ROBINS-I: a tool for assessing risk of bias in non-randomised studies of interventions. BMJ. (2016) 355:i4919. doi: 10.1136/bmj.i4919 27733354 PMC5062054

[85] Higgins JP Morgan RL Rooney AA Taylor KW Thayer KA Silva RA . A tool to assess risk of bias in non-randomized follow-up studies of exposure effects (ROBINS-E). Environ Int. (2024) 186:108602. doi: 10.1016/j.envint.2024.108602 38555664 PMC11098530

[86] Cohen J . A coefficient of agreement for nominal scales. Educ psychol Measurement. (1960) 20:37–46. doi: 10.1177/001316446002000104

[87] Landis JR Koch GG . The measurement of observer agreement for categorical data. Biometrics. (1977) 33:159–74. doi: 10.2307/2529310 843571

[88] Wickham H Averick M Bryan J Chang W McGowan L François R . Welcome to the tidyverse. J Open Source Software. (2019) 4:1686. doi: 10.21105/joss.01686

[89] Broatch JE Dietrich S Goelman D . Introducing Data Science Techniques by Connecting Database Concepts and dplyr. J Stat Educ. (2019) 27(3):147–53. doi: 10.1080/10691898.2019.1647768

[90] Villanueva RAM Chen ZJ . ggplot2: Elegant graphics for data analysis (2nd ed.). Measurement: Interdisciplinary research and perspectives. (2009) 17(3):160–7. doi: 10.1080/15366367.2019.1565254

[91] McKinney W . pandas: a foundational Python library for data analysis and statistics. Python for high performance and scientific computing. (2011) 14(9):1–9.

[92] Hunter JD . Matplotlib: A 2D graphics environment. Computing Sci Engineering. (2007) 9:90–5. doi: 10.1109/MCSE.2007.55

[93] Viechtbauer W . Conducting meta-analyses in R with the metafor package. J Stat Software. (2010) 36:1–48. doi: 10.18637/jss.v036.i03

[94] Benjamini Y Hochberg Y . Controlling the false discovery rate: A practical and powerful approach to multiple testing. J R Stat Society: Ser B (Methodological). (1995) 57:289–300. doi: 10.1111/j.2517-6161.1995.tb02031.x

[95] Schwarzer G . meta: An R package for meta-analysis. R news. (2007) 7:40–5.

[96] Fisher Z Tipton E . robumeta: An R-package for robust variance estimation in meta-analysis. arXiv preprint arXiv:150302220. (2015).

[97] Higgins JPT Thomas J Chandler J Cumpston M Li T Page MJ . Cochrane Handbook for Systematic Reviews of Interventions version 6.5 (updated August 2024). Cochrane (2024). Available online at: https://training.cochrane.org/handbook.

[98] Cuijpers P Griffin JW Furukawa TA . The lack of statistical power of subgroup analyses in meta-analyses: a cautionary note. Epidemiol Psychiatr Sci. (2021) 30:e78. doi: 10.1017/S2045796021000664 34852862 PMC8679832

[99] Malta ES Dutra YM Broatch JR Bishop DJ Zagatto AM . The effects of regular cold-water immersion use on training-induced changes in strength and endurance performance: A systematic review with meta-analysis. Sports Med. (2021) 51:161–74. doi: 10.1007/s40279-020-01362-0 33146851

[100] Xiao F Kabachkova AV Jiao L Zhao H Kapilevich LV . Effects of cold water immersion after exercise on fatigue recovery and exercise performance–meta analysis. Front Physiol. (2023) 14:1006512. doi: 10.3389/fphys.2023.1006512 36744038 PMC9896520

[101] Zhang Y Fan S Hui H Zhang N Li J Liao L . Privacy protection for open sharing of psychiatric and behavioral research data: ethical considerations and recommendations. Alpha Psychiatry. (2025) 26:38759. doi: 10.31083/AP38759 40110382 PMC11915712

