# Supplementary material for: Protocol for a systematic review and meta-analysis on the effects of cold-water exposure on mental health
Source: Front Psychiatry. 2025 Jun 2;16:1603700. doi: 10.3389/fpsyt.2025.1603700 (PMC12171292; doi:10.3389/fpsyt.2025.1603700)
Supplement: Supplementary file 1 [file DataSheet1.pdf]

Supplementary Table 1: Search Strategy

Database: Ovid MEDLINE	
Concept: Cold-water exposure	
**Controlled vocabulary**	**Free-text terms**
1 exp Cold Temperature/	6 Cold
2 exp Cryotherapy/	6 Arctic
4 exp Water/	6 Ice
10 exp animals/	6 Winter
10 not humans.sh.	6 Bath*
	6 Immersion*
	6 Shower*
	6 Swim*
	8 Wim Hof*

Ovid MEDLINE Search Strategy (adapted for other databases as needed)

Ovid MEDLINE(R) ALL <1946 to March 25, 2025>

1 exp Cold Temperature/

2 exp Cryotherapy/

3 1 or 2

4 exp Water/

5 3 and 4

6 ((cold or arctic or ice or winter) adj3 (bath* or immersion* or shower* or swim*)).ab,kf,ti.

7 5 or 6

8 Wim Hof*.ab,kf,ti.

9 7 or 8

10 exp animals/ not humans.sh.

11 9 not 10
